# Blood levels of the micronutrient zinc decrease with advancing age in normally nourished elderly persons

**DOI:** 10.1186/1753-6561-6-S3-P48

**Published:** 2012-06-01

**Authors:** Michiyo Takahashi, Ayumi Takagi, Toshikazu Suzuki, Hikaru Matsumoto

**Affiliations:** 1 Graduate School of Human Ecology, Wayo Women’s University, Ichikawa, Chiba 272-8533, Japan; 2 Department of Health and Nutrition, Wayo Women’s University, Ichikawa, Chiba 272-8533, Japan

## Background

The prevalence of malnutrition in elderly populations has emerged as a social issue. Malnutrition can cause significant deterioration of health, impede individuals’ independence and quality of life, and increase morbidity and mortality rates. Early detection and prompt treatment are very important in preventing malnutrition. The Mini Nutritional Assessment (MNA^®^) is now widely used for nutrition screening and as an assessment tool for elderly persons aged over 65 years. In this study, we investigated which blood nutrients were liable to decrease with advancing age in healthy elderly persons who were classified as normally nourished.

## Materials and methods

The participants comprised 13 elderly adults (7 men and 6 women, mean age 82.5±5.2 years), belonging to a senior citizens’ club in Ichikawa City, Japan. An individual’s physical composition was analyzed using anthropometric parameters. The Nutrition status Mini Nutritional Assessment comprising 18 questions (the full MNA^®^) and a brief-type self-administered diet history questionnaire (BDHQ) were used to evaluate the nutritional status and dietary intake of subjects, respectively. The levels of various nutrients in the blood were determined by laboratory biochemical blood tests. Measurements were performed in December 2011 and the data were compared with data obtained from the same participants two and half years ago (March 2009) [1].

## Results

The average screening score of the full MNA^®^ from March 2009 and December 2011 was 26.7 ± 1.3 and 27.5 ± 1.3, respectively. The estimated nutrient intake measured by BDHQ analysis revealed all participants were well nourished. The Body Mass Index score from December 2011 (23.0 ± 1.8) was slightly but significantly lower (*p*<0.01) than that from March 2009 (23.5 ± 2.0). Although the concentration of red blood cells, hematocrit, hemoglobin content, and serum levels of total protein, albumin, and iron were not significantly different compared with the original measurements (two years and nine months previously), these values appeared to be declining. However, the levels of serum zinc had decreased by 22% during the last 2 years 9 months (*p*<0.001), although levels were still within the normal range.

## Conclusions

The results presented here suggest that elderly people might become susceptible to zinc insufficiency with increasing age, even if they are considered well-nourished. Zinc deficiency causes significant impairment of both the adaptive and innate immune responses, and is important for promoting systemic inflammation [2]. Therefore, it is important for nutritionists to monitor the consumption of micronutrients such as zinc more carefully, in order to maintain and enhance the quality of life in elderly people.
